# Systemic mesenchymal stem cell therapy for reduction of inflammatory burden in a patient with juvenile-onset rheumatoid arthritis and dialysis-dependent renal failure

**DOI:** 10.3325/cmj.2026.67.238

**Published:** 2026-06

**Authors:** Dragan Primorac, Petar Brlek, Luka Bulić, Miomir Knežević, Gordana Kalan Živčec

**Affiliations:** 1 St. Catherine Specialty Hospital, Zagreb, Croatia; 2 International Center for Applied Biological Research, Zagreb, Croatia; 3 School of Medicine, Josip Juraj Strossmayer University of Osijek, Osijek, Croatia; 4 Faculty of Dental Medicine and Health, Josip Juraj Strossmayer University of Osijek, Osijek, Croatia; 5 Eberly College of Science, The Pennsylvania State University, State College, PA, USA; 6 School of Medicine, University of Split, Split, Croatia; 7 The Henry C. Lee College of Criminal Justice and Forensic Sciences, University of New Haven, New Haven, CT, USA; 8 Sana Kliniken Oberfranken, Coburg, Germany; 9 School of Medicine, University of Rijeka, Rijeka, Croatia; 10 School of Medicine, University of Pittsburgh, Pittsburgh, PA, USA; 11 Gandhinagar Campus, National Forensic Sciences University, Gandhinagar, India; 12 Department of Molecular Biology, Faculty of Science, University of Zagreb, Zagreb, Croatia; 13 Algebra Bernays University, Zagreb, Croatia; 14 GaiaCell, Advanced Cell and Gene Therapy Ltd., Trzin, Slovenia

## Abstract

Chronic systemic inflammation is a key driver of disease progression in rheumatoid arthritis and chronic kidney disease (CKD), particularly in patients undergoing long-term hemodialysis. Persistent elevation of proinflammatory cytokines contributes to pain, anemia, endothelial dysfunction, and increased cardiovascular risk, while therapeutic options are often limited by comorbidities and drug intolerance. Mesenchymal stem cells (MSCs) possess immunomodulatory properties that may offer an alternative strategy for systemic inflammatory control. We report on the case of a 56-year-old woman with juvenile-onset rheumatoid arthritis and dialysis-dependent CKD who received three consecutive systemic administrations of MSCs. Baseline evaluation demonstrated elevated inflammatory markers, including C-reactive protein, interleukin-6, and tumor necrosis factor-α. Following treatment, inflammatory parameters were consistently reduced, which was accompanied by improvement in hemoglobin levels and favorable trends in renal function markers. Additionally, pain assessment scores (Western Ontario and McMaster Universities Arthritis Index, Knee Injury and Osteoarthritis Outcome Score, Visual Analogue Scale) showed clinically significant improvement. The patient also reported significant subjective pain relief and improved overall well-being. Although limited by its single-case design, this report supports the biological plausibility of systemic MSC therapy as a potential immunomodulatory approach in patients with overlapping autoimmune and uremia-associated chronic inflammation. Further controlled studies are warranted.

Chronic systemic inflammation is a hallmark of autoimmune and inflammatory rheumatic diseases and represents a key driver of progressive tissue damage, persistent pain, and multisystem complications. In juvenile rheumatoid arthritis (JRA), sustained immune dysregulation leads to synovial hyperplasia, cartilage destruction, and bone erosion, mediated by proinflammatory cytokines such as tumor necrosis factor-α (TNF-α), interleukin-1 (IL-1), and IL-6, and other proinflammatory markers such as C-reactive protein (CRP) and procalcitonin. Beyond local joint pathology, these mediators exert systemic effects, contributing to endothelial dysfunction, anemia of chronic disease, metabolic disturbances, and increased cardiovascular risk (1). Another factor in chronic inflammatory arthropathies is pain, which is usually multifactorial. While mechanical factors related to joint destruction and altered biomechanics play a significant role, particularly in patients requiring arthroplasty, persistent inflammatory signaling amplifies peripheral and central sensitization pathways (2). Cytokine-mediated activation of nociceptors lowers pain thresholds and sustains hyperalgesia, while chronic inflammation may promote maladaptive neuroimmune interactions within the central nervous system. Consequently, pain severity does not always correlate directly with radiological damage, which underscores the importance of addressing the inflammatory component even in advanced disease stages (3).

Systemic inflammation is also closely intertwined with chronic kidney disease (CKD), particularly in patients receiving long-term hemodialysis. CKD is increasingly recognized as a state of persistent low-grade inflammation driven by uremic toxin accumulation, oxidative stress, endothelial activation, altered gut permeability, and repeated exposure to dialysis membranes (4). Elevated circulating inflammatory markers, such as CRP, IL-6, and TNF-α, are frequently observed in dialysis-dependent patients and are associated with increased cardiovascular morbidity, anemia, protein-energy wasting, and impaired immune regulation (5). In such individuals, systemic inflammation may be both a consequence of renal failure and an independent contributor to disease progression and symptom burden. Importantly, chronic inflammation in CKD may exacerbate pain perception and fatigue, further reducing functional capacity in patients already burdened by severe musculoskeletal pathology. Therapeutic options for controlling inflammation in this population are limited, as the use of nonsteroidal anti-inflammatory drugs (NSAIDs) requires extreme caution due to nephrotoxicity and gastrointestinal risk (6). On the other hand, long-term corticosteroid therapy is associated with metabolic complications, immunosuppression, and increased cardiovascular risk (7).

Mesenchymal stem cells (MSCs) have emerged as a promising therapeutic approach due to their potent immunoregulatory and anti-inflammatory properties. Initially investigated for their regenerative capacity, MSCs are now understood to function predominantly through paracrine and immunomodulatory mechanisms rather than through direct tissue replacement (8). They interact dynamically with components of both the innate and adaptive immune systems. MSCs suppress proliferation and activation of effector T lymphocytes, promote expansion of regulatory T cells, inhibit B-cell maturation and antibody production, and modulate dendritic cell and macrophage differentiation (9).

At the molecular level, MSCs secrete a broad array of bioactive mediators, including transforming growth factor-β, and extracellular vesicles enriched with regulatory microRNAs (10). These factors attenuate cytokine production, downregulate inflammatory cascades, and reduce systemic immune activation. Notably, preclinical and early clinical studies suggest that MSCs may also exert protective and anti-inflammatory effects in renal injury models by modulating immune cell infiltration, reducing fibrosis, and attenuating oxidative stress (11). Although evidence in advanced dialysis-dependent CKD remains limited, the immunomodulatory properties of MSCs provide a biologically plausible rationale for targeting systemic inflammation in this setting.

The patient described in this report had a history of JRA with extensive joint destruction requiring multiple arthroplasties, accompanied by chronic renal insufficiency secondary to chronic glomerulonephritis and long-term hemodialysis. In this clinical context, systemic inflammation likely reflected the combined effects of autoimmune disease and uremia-related immune dysregulation. Given the limitations of conventional anti-inflammatory therapies and the potential contribution of systemic inflammation to persistent pain and functional impairment, a systemic immunomodulatory strategy was considered. Three consecutive systemic administrations of mesenchymal stem cells were performed with the primary aim of reducing systemic inflammatory activity and, secondarily, alleviating pain and improving clinical status.

## Case report

### Patient history

A 56-year-old female patient had a longstanding history of JRA, diagnosed at the age of four. Over the years, she was treated with corticosteroids, NSAIDs, azathioprine, and gold salts. The disease course was complicated by severe degenerative joint changes requiring multiple orthopedic interventions. She underwent total hip arthroplasty of the left hip in 1996, bilateral total knee arthroplasties in 2014, and reconstruction of the ankle joints in 2015. In 2017, she underwent incision and drainage of the right trochanteric region, when aseptic necrosis of the right femoral head was confirmed.

She also developed chronic renal insufficiency secondary to chronic glomerulonephritis, and she has been on maintenance hemodialysis since 2016. In 2016, she developed a hematoma at the site of a vascular extraction procedure, requiring surgical removal of a coagulum and subsequent antibiotic therapy. During the initial period of dialysis, she experienced episodes of chills, rigors, and fever, which have since resolved. The same year, she was hospitalized for erosive gastritis associated with NSAID use. In 2017, she underwent cholecystectomy due to cholelithiasis.

In 2015, the patient experienced a syncopal episode preceded by generalized weakness and profuse sweating. The episode was not accompanied by convulsions or tongue biting, although urinary incontinence was reported. Brain magnetic resonance imaging (MRI) was normal. Electroencephalography (EEG) was performed on three occasions; the initial recording showed paroxysmal activity, after which lamotrigine therapy was introduced. The patient subsequently discontinued antiepileptic therapy, and follow-up EEG findings were normal. A tilt-table test reportedly yielded pathological findings. Since then, she has not reported recurrent convulsive episodes.

Approximately 10-15 days before one of her outpatient evaluations, the patient developed high-frequency bilateral tinnitus, which she described as highly disturbing. She also reported subjective hearing impairment and episodes of low blood pressure, which she believed exacerbated the tinnitus. She denied acute systemic illness at that time. Videoendoscopic examination of the left ear revealed crusting and cerumen accumulation deep in the external auditory canal; after removal, a small amount of purulent discharge was noted. The right ear examination was unremarkable. Cranial MRI performed previously was normal.

At the time of neurological examination, the patient was conscious, fully oriented, and with normal speech. Cardiovascular and respiratory status was compensated. Gait was impaired due to right hip pain, with dragging of the right leg. Multiple degenerative deformities of both small and large joints were evident in the context of longstanding RA. A right conjunctival hematoma was noted; the patient reported frequent spontaneous bruising elsewhere on the body. Both knees were edematous, more pronounced on the right. Global muscle strength was preserved within the available range of motion. She reported no sensory disturbances. Cranial nerves were intact, and Romberg testing was negative.

### Medical intervention

The protocol was designed to ensure a controlled therapeutic environment while adhering to established principles of regenerative medicine and advanced cell therapy safety standards (GaiaCell, Advanced Cell and Gene Therapy Ltd, Slovenia; Oct, 2025). Particular attention was given to dose calculation, cell preparation, infusion procedures, and patient monitoring. MSCs were administered through systemic intravenous infusion, which represents one of the most frequently used delivery routes in clinical MSC-based therapies. Intravenous administration allows the cells to circulate through the systemic vasculature and interact with immune cells and injured tissues, while also enabling relatively simple and minimally invasive delivery.

The dose was calculated based on the patient’s body weight, following dosing strategies commonly reported in clinical MSC research. A dose of 1.2 × 10^6^ MSC per kilogram of body weight was selected for the intervention. Weight-adjusted dosing is widely used in cell therapy to ensure proportional exposure of the patient’s physiological systems to the administered cellular product. Based on the patient’s body weight, the calculated therapeutic dose corresponded to approximately 70 million MSCs. The final dose consisted of the combination of two separate preparations containing 50 million and 20 million MSCs, respectively.

The infusion procedure was conducted within a controlled clinical environment supplied with appropriate monitoring equipment and emergency medical response capabilities. The total infusion volume was restricted to a maximum of 200 mL, including both the cellular suspension and the carrier solution. Vital physiological parameters were monitored throughout the entire infusion procedure. These included blood pressure, heart rate, oxygen saturation, and respiratory status. Following completion of the infusion, the patient remained within the clinical facility for a post-infusion observation period of approximately 30 minutes. The purpose of this observation period was to monitor for any immediate adverse reactions. During this time, health care personnel continued to assess the patient’s vital signs and overall clinical condition.

The patient was transferred to and from the treatment facility by an ambulance. The presence of trained medical personnel during transport ensured that appropriate medical assistance could be provided if unexpected complications occurred during transit.

**Figure 1:**
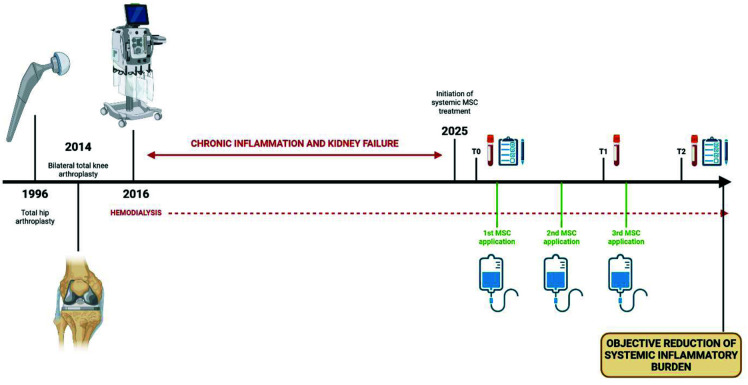
Complete clinical timeline (created with Biorender.com). MSC – mesenchymal stem cells.

The therapeutic intervention included three systemic MSC applications, approximately six weeks apart. Biochemical and inflammatory parameters were determined at three time points: T0 – before the first application, T1 – after the second application, and T2 – after the third application. Additionally, Western Ontario and McMaster Universities Arthritis Index (WOMAC) pain, Knee Injury and Osteoarthritis Outcome Score (KOOS) pain, and Visual Analogue Scale (VAS) scores were determined at T0 and T2. A complete clinical timeline is shown in Figure 1.

### Intervention results

Over the course of systemic MSC application, changes in inflammatory and biochemical parameters were observed (Table 1).

**Table 1:** Biochemical and inflammatory parameters before the first (T0), after the second (T1), and after the third mesenchymal stem cell application (T2)

Parameter	T0	T1	T2	Reference interval
Sedimentation rate (mm/3.6 ks)	20	6	2	5-28
C-reactive protein (mg/L)	30	3.1	3.1	<5.0
Hemoglobin (g/L)	114	131	133	119-157
Troponin I (ng/L)	84.6	55.9	65.1	<15.6
Aspartate aminotransferase (U/L)	13	12	15	8-30
Alanine aminotransferase (U/L)	10	17	23	10-36
Gamma-glutamyl transferase (U/L)	31	23	38	9-35
Alkaline phosphatase (U/L)	160	120	132	64-153
Amylase (U/L)	106	92	101	28-100
Creatinine (μmol/L)	245	194	179	49-90
Urea (mmol/L)	7.7	7.1	9.0	2.8-8.3
Urate (μmol/L)	180	170	160	134-337
Glomerular filtration rate CKD-EPI (mL/min/1.73 m^2^)	18	24	27	G1: >89 G2: 60-89 G3a: 45-59 G3b: 30-44 G4: 15-29 G5: <15
Sodium (mmol/L)	140	139	142	137-146
Potassium (mmol/L)	5.0	4.0	4.3	3.9-5.1
Calcium (mmol/L)	2.17	2.30	2.34	2.14-2.53
Iron (μmol/L)	5	6	7	8-30
Unsaturated iron binding capacity (μmol/L)	22	21	25	26-59
Total iron-binding capacity (μmol/L)	27	27	32	49-75
Vitamin B12 (pmol/L)	457	386	643	138-652
Folate (nmol/L)	89.4	>90.6	>90.6	7.0-46.4
Vitamin D (nmol/L)	42	27	24	>75
Procalcitonin (μg/L)	0.47	0.81	1.19	<0.10
Interleukin (IL)-1β (pg/mL)	<16.0	<16.0	<16.0	<16.0
IL-2 (pg/mL)	<2.6	<7.0	<7.0	<5.0
IL-6 (pg/mL)	34.3	29.1	13.1	<5.9
IL-10 (pg/mL)	5.4	N/A	<5.0	<10.8
Tumor necrosis factor-α (pg/mL)	13.8	11.9	8.4	<12.0

**Figure 2:**
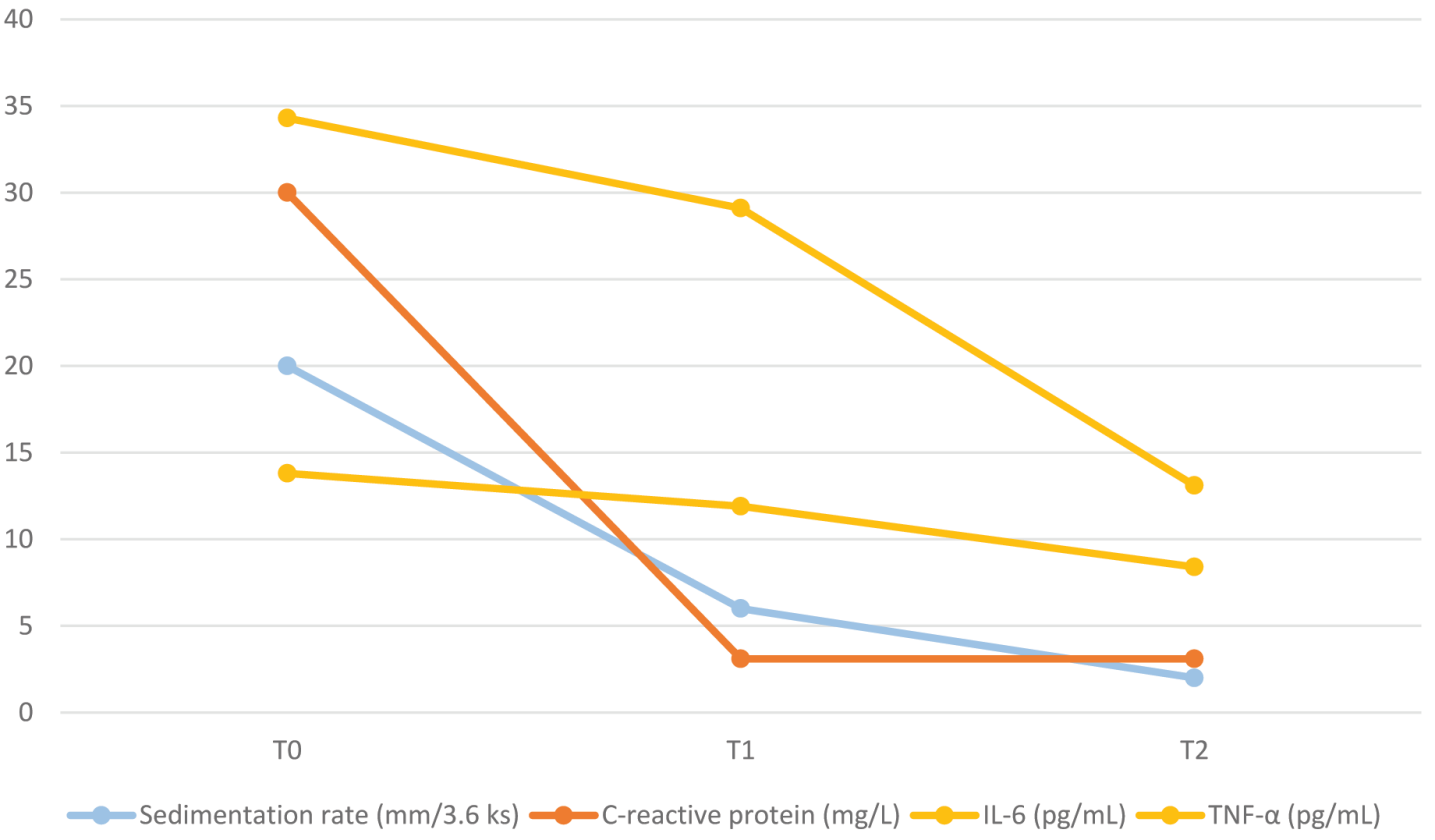
Observed anti-inflammatory trends across time points. T0 – before the first mesenchymal stem cells application; T1 – after the second application; T2 – after the third application; IL – interleukin; TNF – tumor necrosis factor-α.

An anti-inflammatory trend was observed, as indicated by a decreasing erythrocyte sedimentation rate, C-reactive protein, IL-6, and TNF-α values (Figure 2).

Contrary to these trends, the values of procalcitonin steadily increased through the three time points.

Positive trends in terms of end-organ function were observed during the course of treatment, as reflected by changes in key laboratory parameters. A progressive decrease in troponin I levels suggested a reduction in myocardial stress or injury, indicating potential improvement in cardiac status. At the same time, serum creatinine concentrations declined, accompanied by a corresponding increase in the estimated glomerular filtration rate (eGFR). These findings point toward an improvement in renal function and suggest a possible attenuation of systemic inflammatory or metabolic burden affecting kidney performance.

In terms of quantitative pain assessment, clinically significant changes (greater than 15%) were observed in WOMAC pain, KOOS pain, and VAS scores between T0 and T2 (Table 2).

**Table 2:** Pain assessment scores before the first (T0) and after the third mesenchymal stem cell application (T2)

Pain assessment score	T0	T2
Western Ontario and McMaster Universities Arthritis Index pain^†^	100%	25%
Knee Injury and Osteoarthritis Outcome Score pain^*^	14%	79%
Visual Analogue Scale	8	1

*normalized.

In addition to these quantitative biochemical and clinical improvements, the patient also experienced notable clinical benefits. Following the second treatment, the patient reported a marked improvement in overall subjective well-being, describing increased energy levels and a general sense of recovery. Importantly, perceived pain intensity was significantly reduced, which had previously been one of the dominant symptoms limiting daily functioning. This improvement in pain perception was consistent with the observed biochemical trends and may reflect the systemic immunomodulatory and anti-inflammatory effects of the administered therapy. Together, these findings suggest that the intervention may have contributed to both measurable physiological improvements and meaningful symptomatic relief.

## Discussion

This case illustrates a potential role of systemic MSC therapy as an immunomodulatory strategy in a patient with overlapping autoimmune and uremia-associated chronic inflammation. Following three systemic MSC applications, key inflammatory markers were consistently reduced, which was accompanied by subjective pain improvement and favorable trends in selected end-organ parameters.

The anti-inflammatory pattern was most evident in a marked decrease in erythrocyte sedimentation rate (20 → 6 → 2 mm/3.6 ks) and CRP (30 → 3.1 → 3.1 mg/L), as well as in IL-6 (34.3 → 29.1 → 13.1 pg/mL) and TNF-α (13.8 → 11.9 → 8.4 pg/mL). These findings are biologically coherent with the established immunomodulatory properties of MSCs. Experimental and clinical data suggest that MSCs attenuate systemic inflammation primarily through paracrine signaling rather than direct engraftment, modulating both innate and adaptive immune responses (12). Suppression of proinflammatory cytokine production, promotion of regulatory T-cell expansion, and polarization of macrophages toward an anti-inflammatory phenotype provide a plausible mechanistic explanation for the observed biomarker shifts in this patient.

Importantly, the clinical context in this case extends beyond isolated autoimmune inflammation. CKD, particularly in patients undergoing long-term hemodialysis, is recognized as a state of persistent low-grade systemic inflammation. Uremic toxin accumulation, oxidative stress, complement activation by dialysis membranes, and recurrent vascular access interventions all contribute to chronic immune activation. Elevated CRP and IL-6 levels in dialysis patients are associated with cardiovascular morbidity and mortality (13). Therefore, the reduction of inflammatory biomarkers observed after MSC administration may reflect modulation of both RA-related and uremia-associated inflammatory pathways. This dual inflammatory burden likely amplified the patient’s symptomatology, including chronic pain and fatigue, making systemic immunoregulation particularly relevant.

The improvement in hemoglobin (114 → 131 → 133 g/L) may also be interpreted within the inflammatory framework. Anemia of chronic disease is mediated in part by IL-6-induced hepcidin upregulation and impaired iron utilization (14). The decline in IL-6 levels after MSC therapy may have contributed to improved erythropoiesis or iron handling, although this hypothesis cannot be confirmed in a single case. Similarly, the observed decrease in creatinine (245 → 194 → 179 μmol/L) and increase in estimated glomerular filtration rate (18 → 24 → 27 mL/min/1.73 m^2^) are intriguing. While dialysis-dependent CKD typically limits the interpretation of eGFR changes, these trends raise the possibility that systemic inflammatory attenuation may positively influence renal hemodynamics or residual renal function. Preclinical studies suggest that MSCs can reduce renal inflammation and fibrosis, but robust evidence in advanced CKD remains limited (15). Therefore, these findings should be interpreted cautiously and primarily as hypothesis-generating.

The patient reported a significant subjective reduction in pain and improvement in overall well-being after the third MSC application. Chronic inflammatory pain is driven not only by structural joint damage but also by cytokine-mediated sensitization of peripheral nociceptors and central pain pathways. The parallel reduction in IL-6 and TNF-α, both implicated in pain amplification, supports a potential mechanistic link between systemic cytokine modulation and symptomatic relief (16,17). Although no standardized pain scale was reported, the temporal association between biomarker decline and symptom improvement strengthens the plausibility of a biologically mediated effect.

One notable finding was a progressive increase in procalcitonin levels (0.47 → 0.81 → 1.19 μg/L), despite the overall anti-inflammatory trend. In dialysis patients, procalcitonin can be chronically elevated in the absence of overt infection due to reduced renal clearance and persistent low-grade inflammation (18). Moreover, fluctuations may occur in relation to dialysis sessions or subclinical inflammatory stimuli. In the absence of clinical signs of infection and given the concurrent decrease in CRP and proinflammatory cytokines, this isolated biomarker rise should be interpreted with caution. Nonetheless, it underscores the importance of careful infection surveillance when applying systemic cellular therapies in immunologically complex patients.

Pain assessment scores demonstrated a marked and clinically meaningful reduction in pain across all applied assessment instruments between baseline (T0) and follow-up (T2). Specifically, normalized WOMAC pain decreased from 100% to 25%, while KOOS pain improved from 14% to 79%, which indicates substantial functional and symptomatic recovery. In parallel, VAS showed a pronounced reduction from 8 to 1, further supporting a significant decrease in patient-reported pain intensity. Importantly, all observed changes exceeded the commonly accepted threshold for clinical relevance (>15%), underscoring the robustness of the treatment effect. The consistency of improvement across multiple validated pain scales strengthens the reliability of these findings and reduces the likelihood that the observed effects are attributable to measurement bias or instrument-specific variability.

**Figure 3:**
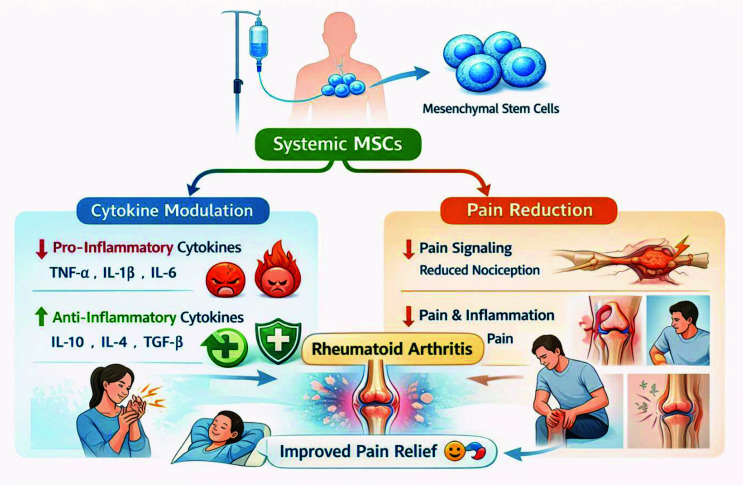
Systemic mesenchymal stem cells (MSC) application effect on inflammation modulation and pain reduction.

This case highlights several clinically relevant considerations. First, systemic MSC therapy may offer a therapeutic avenue for patients in whom conventional anti-inflammatory agents are contraindicated or poorly tolerated. In this patient, NSAID-related erosive gastritis and dialysis-dependent renal insufficiency significantly limited pharmacological options. Second, biomarker-guided monitoring provides an objective framework for evaluating biological response in experimental or compassionate-use interventions. Serial measurement of inflammatory cytokines and acute-phase reactants allowed documentation of a coherent anti-inflammatory trajectory (Figure 3).

However, several limitations must be acknowledged. This is a single case report without a control comparator. Spontaneous fluctuation of inflammatory markers cannot be excluded, particularly in a patient undergoing hemodialysis. The absence of standardized disease activity scores (eg, Disease Activity Score-28 for Rheumatoid Arthritis) or validated pain scales limits quantitative assessment of clinical improvement. Furthermore, the durability of the observed effects remains unknown, as long-term follow-up data are not yet available. The increase in procalcitonin also warrants further investigation in future applications to better characterize safety signals.

In summary, this case suggests that systemic administration of MSC may reduce inflammatory burden in a patient with complex multimorbidity characterized by JRA and dialysis-dependent CKD. The observed decline in CRP, IL-6, TNF-α, and erythrocyte sedimentation rate, together with subjective pain improvement and favorable trends in selected organ-function parameters, supports the biological plausibility of MSC-mediated immunomodulation in overlapping autoimmune and uremic inflammation. While definitive conclusions cannot be drawn from a single case, these findings encourage further controlled studies exploring systemic MSC therapy as a potential adjunctive strategy in patients with refractory inflammatory states and limited therapeutic options.
